# Adding Value to Secondary Aluminum Casting Alloys: A Review on Trends and Achievements

**DOI:** 10.3390/ma16030895

**Published:** 2023-01-17

**Authors:** Helder Nunes, Omid Emadinia, Rui Soares, Manuel F. Vieira, Ana Reis

**Affiliations:** 1 LAETA/INEGI, Institute of Science and Innovation in Mechanical and Industrial Engineering, 4200-465 Porto, Portugal; 2 Faculty of Engineering, University of Porto, 4200-465 Porto, Portugal

**Keywords:** secondary aluminum alloys, recycling, intermetallic compounds, microstructure

## Abstract

Aluminum is a critical element of the circular economy as it can be recycled several times. Moreover, Al recycling is a more economically and environmentally efficient procedure than the primary Al production from ores. Secondary aluminum alloys are mostly used in casting applications since it is possible to accommodate their chemical composition through secondary manufacturing processes. However, the quality of the alloys may be considerably altered during the different steps of the recycling process. Inadequate waste sorting might result in excessive contamination. Iron is the most dangerous contaminant because it causes brittle and fragile intermetallic phases, which significantly impacts the mechanical characteristics of alloys. In addition, the microstructure of the alloy changes significantly after multiple cycles of remelting. These issues lead to the downcycling of aluminum, i.e., in other words, the reduction in the overall quality of the alloys. Thus, it has been shown that a number of procedures, including ultrasonic melt treatment and microalloying with rare earths, can somewhat alter the shape of the Fe-rich phases in order to reduce the shortcomings of downcycling. However, a solid mechanical characterization is still missing in order to improve the Fe-rich phase alteration.

## 1. Introduction

Due to the ability of aluminum (Al) to be recycled several times without major losses of some original properties, such as formability and lightness, it is considered a circular material and is vital for a climate-neutral and circular economy. Nowadays, 36% of aluminum production focuses on recycling aluminum, also known as secondary production. Moreover, by 2050, this value can reach 50% of the European Union demand and prevent 39 million tons of CO_2_ emissions per year. The promotion of secondary production can also reduce the EU dependency on other countries for Al, such as China, which produces more than 55% of all primary Al [1,2].

The two types of Al production, primary and secondary, vary on the raw material used and processes needed to convert this into its usable Al in further manufacturing processes. The primary production of Al uses Al-rich ores, mainly bauxite, while secondary production uses scraps and other residues. Primary production presents various disadvantages, such as high greenhouse gas emissions and energy-intensive needs [3,4,5]. However, the primary driver of interest in secondary manufacturing is linked to the economic benefits [6,7]. Original manufacturing requires 180 MJ/kg of energy, whereas secondary production only requires 15 MJ/kg, representing about a 90% reduction in energy consumption [8,9]. The energy difference is due to fact that the raw production of materials, with bauxite in the Bayer process, needs high temperatures (373 and 593 K) and pressures (30 atm) to be reduced into an oxide (Al_2_O_3_) through different stages, such as clarification, precipitation, and calcination [3,10,11]. Afterwards, the Hall–Heroult process is performed to convert Al_2_O_3_ into molten aluminum. This electrolytic extraction process also needs large amounts of electricity to achieve the high temperatures needed, between 950 °C and 980 °C, and to apply a direct current to the molten solution. Thus, the total energy consumption from bauxite reduction to Al ingots production is about 168,000 MJ/ton of aluminum [9].

The major procedure of secondary Al production involves the remelting of scraps, by-products, and end-of-life products into secondary aluminum alloys (SAAs). The anticipated rise in Al waste has also sparked significant interest in recycling. Al components have a finite lifespan that ranges from 0.2 years for beverage cans to 15 years for automotive parts and 50 years for extruded components used in construction [12,13]. Furthermore, due to various factors such as different alloy mixing and contaminations, mainly of Fe, during the recycling process, the SAAs tend not to present the same mechanical properties as the primary alloys, thus limiting the application range of SAAs and their value. SAAs play a large role in the automotive sector, particularly in high-pressure die casting (HPDC), and typical AlSiCu(Fe) alloys are considered for around 70% of the utilized alloys. Thus, their reusage is advantageous from a practical, financial, and environmental perspective [14,15].

The Al industry for processing SAAs, whether conventional or advanced alloys, requires new techniques to enhance the overall quality and applicability of these alloys. The conventional recycling process is economically favorable for the near future but, with the increase in the scrap availability including advanced Al alloys and the use of additions such as Si to enhance the fluidity, new methodologies should thus be developed. Some of the most recent advancements focus on the partial or total recovery of pure aluminum, such as fractional crystallization [16]. In consideration of the currently used cycles of component production, the automotive industries need to maintain economic competitive edge by lowering the manufacturing costs. This review article aims to analyze the current studied techniques of SAA valorization by modifying the Fe-rich phases present in the alloys that might be easily applied in the manufacture process without excessive costs to the manufactures. Thus, this article aims to comprehend the forecasts of SAA’s future evolution and discuss the Al recycling process and its limits. Furthermore, newer studies that aim to improve and add value to SAAs are finally discussed.

## 2. Materials and Methods

The state of the art to construct this study was obtained from Web of Science (WoS) databases until the end of October 2022. Aluminum casting alloys, secondary alloys, recycling of aluminum, remelting, downcycling, microalloying, ultrasonic melt treatment, and other terminology were employed as the searching keys. However, some papers obtained from this research were not considered for this study, such as beverage can recycling or non-casting recycling methodologies such as extrusion. Thus, this study focuses on the processing of SAAs for industrial applications that require adequate mechanical properties alongside microstructural analyses.

After reviewing the majority of the papers, the major aspects related to the issues and challenges of SAAs were identified as: potential alloy microstructural alterations and contaminations during the recycling process; the phenomena of quality loss in the recycled alloys; and the documented attempts at lowering the effects of Fe-rich phases in the alloys. These were roughly translated into the following various sections of the article:

- Section 3—Aluminum Recycling Process: The primary focus of this section is to both comprehend the recycling cycle that generates the SAA casting alloys and highlight the key factors in each phase that may cause an alloy to deteriorate in quality and properties.
- Section 4—Aluminum Downcycling: This section emphasizes how the downgrade of aluminum fits into the three major downcycling aspects: functional, thermodynamic, and economic.
- Section 5—The Microstructure and Mechanical Performance of SAAs: Since the microstructure and mechanical properties of commercially used alloys have a close relationship to the alloy quality loss, this section describes the typical microstructure, chemical composition, and mechanical properties of some of the most used SAA alloys.
- Section 6—Valorization of SAAs: This section mainly focuses on strategies that attempt to lessen the effects of Fe-rich phases by modifying their phase and/or morphologies out of all the reported breakthroughs in techniques to add value to the SAA alloys. Emphasis is also placed on techniques that are simple to include into the often-utilized component manufacture cycles, such melt treatment or heat treatment.

## 3. Aluminum Recycling Process

The producers of SAAs can be divided into two major groups—remelters and refiners. The former group produces typically wrought alloys in billets or slabs by remelting clean and well-sorted scraps. On the other hand, the refiners produce common casting alloys from all types of scraps. In this case, the production of SAAs not only consists of collection, sorting, and remelting, but also has a final step of refining before casting [17].

### 3.1. Collection and Sorting

The initial stage in recycling is to gather scraps from various sources, including fabrication facilities, scrap collectors, and sellers. Specific processes are often used to obtain the Al from some of these scraps. For instance, it was necessary to disassemble end-of-life cars to retrieve the mono-material components before shredding the components made of several materials. To remove other materials fixed to the aluminum, the size of the aluminum scrap is crucial. The needed size typically ranges from 100 to 150 mm; thus, shredding is an important step [9].

Scrap is sorted using various methods, including magnetic separation to remove iron and steel, Eddy current separation to separate non-metallic particles, and sink float separation to remove loose non-Al components. The magnetic separation can remove particles smaller than 10 mm by using a magnetic drum separator and a vibrating screen. The magnetic field produced by the Eddy current separator momentarily transforms metallic particles into “magnets” with their poles aligned. These particles are then repulsed and ejected with a different ballistic curve than the non-metallic particles. The final method, sink float separation, uses a liquid with greater density than aluminum. When the scraps are added to the liquid, aluminum scraps float to the top while loose particles sink [18,19].

There is now little economic incentive to invest in more advanced scrap sorting systems or to use post-consumer scrap more widely. Future primary aluminum consumption might be reduced by 15–25% if scrap sorting is applied on end-of-life cars if the scrap collection rate is improved in the future, mainly through political measures [20,21]. Additionally, by using more advanced technologies such as laser-induced breakdown spectroscopy (LIBS) with classification algorithms and machine learning techniques, scrap sorting could become a preventive strategy to avoid contamination and downcycling. LIBS employs a focused pulse laser to remove just a minuscule portion of the surface material. This material quickly reaches a temperature of 10,000 °C, forming plasma. A spectrometer then processes the element-specific light released during this plasma’s cooling. Each element emits a distinct number of wavelengths, resulting in spectra with thousands of peaks. Finally, classification algorithms may be used to analyze the spectra obtained from LIBS, and the concentration may be computed from the peak intensity [22,23].

With the scraps adequately sorted, further shearing and baling can be carried out to produce large dense products, known as bales, which are easier to handle than loose scraps. This process compacts the scraps into cubes of about 1 m × 1 m × 1.5 m. Other processes can be performed to the scraps before melting to maximize Al recovery, such as drying to remove moisture and oil contamination and de-coating to eliminate possible paints on the surface of the scraps [17].

### 3.2. Remelting

The remelting of sorted Al scrap is carried out normally in reverberatory or tilt rotary furnaces. The latter is normally used by large-scale secondary smelters in Europe which have a capacity between 2 and 20 tons. In this technique, the Al scraps and other chemical elements needed to obtain a specific chemical composition, such as Si or Cu, are loaded into the refractory-lined chamber with salt flux such as NaCl. The material is then heated up and melted while the chamber rotates. With the material fully molten, this can be transferred to ladles, molds, or direct chill casts [17,24].

**Figure 1:**
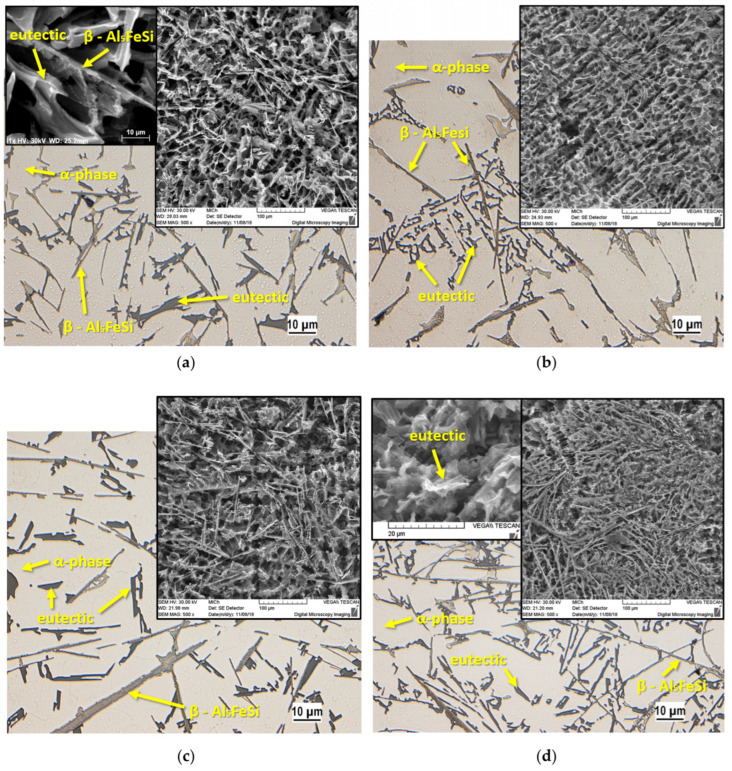
Microstructures of the AlSi9Cu alloy after: (**a**) one cycle of remelting, (**b**) three cycles of remelting, (**c**) five cycles of remelting, and (**d**) seven cycles of remelting [27].

One of the sources of impurities, microstructure changes, and eventual reductions in applicability is the repeated remelting of the material. Not only do some alloying elements, such as eutectic Si modifiers including Sr, tend to be lost, but also the contents of some impurities tend to rise after numerous remelting stages. Several studies examine the effects of up to seven remelting cycles on the microstructure of alloys. These showed several alterations, such as the coarsening of grains, the unevenness of dendritic cells, increased porosity, the coarsening of intermetallic phases, and eutectic Si modification regression [6,25,26,27]. For instance, Kasińska et al. [27] assessed the impacts of increasing the returnable material on the homogeneity and susceptibility of castings to investigate defect formations such as hot tearing, in addition to studying the effects of several cycles of remelting the AlSi9Cu alloy. The researcher noticed that the tensile strength and elongation had declined by the fourth cycle of remelting. The main cause of this loss of properties is attributed to the rise in Fe content due to the contamination of steel crucibles (the Fe content increased from 1.41 to 1.61 wt.% by the seventh cycle). The formation of needle-shaped β phases, as shown in Figure 1, confirmed that these phases cause cracking and microporosity. Moreover, these authors revealed that the tensile properties of the alloys that received a higher number of remelting stages (more than five) were enhanced after receiving an artificial ageing heat treatment; this improvement was attributed to the reduction in the size of the β phase (needle-like) and the precipitation of coherent and semi-coherent phases.

One of the main sources of scraps used in the production of SAAs is returnable material, which consists of cast-off pieces such as gating, riser, and venting systems. It is common in the sector to fuse primary alloys with these recovered materials to reduce production costs. However, the quality of the casting tends to decline as the proportion of returnable materials rises caused by contaminations such as oxides and hydrogen that lead to an increase in porosities and other defects. Matejka et al. [28] demonstrated that the microstructure of the HPDC AlSi9Cu3(Fe) alloy was primarily affected by the coarsening of Si particles, particularly in the center area of the casting when returnable material usage was greater than 75%. These authors revealed that, in the as-cast state, no significant impact on porosity levels was observed, but the application of heat treatment provoked an increase in the pore size. In a different study [29], the hot tearing index and other qualitative and quantitative measurements were used to examine the susceptibility to hot tearing. The permissible sensitivity limits for this defect were achieved when the returnable material fraction reached 50% of the bath. Even a 20% returnable material fraction had a negative effect. The researchers reached the conclusion that, in order to maintain high-quality castings, the ideal utilization of returnable material percentage should be about 50%. Usages above 50% of returnable material might be troublesome and are only advised for use in simple-shaped castings or in components that will not be employed under stress.

### 3.3. Refining

The refining process includes the metal-cleaning and degassing stages prior to casting. Several techniques can be used to improve the melt cleanliness, such as flux treatments, degassing, and others [30].

The flux treatment uses salts to clean the molten Al. These are frequently mixtures of NaCl and KCl with various fluorites and chloride additions. These salts are dispersed across the melt surface, halting further oxidation while removing the oxide layers. However, this method generates a hazardous byproduct known as secondary aluminum dross, rich in the metal Al and soluble salts, though it is not commonly used [31,32].

Another technique of degassing involves rotary technology. It requires the use of a graphite rotor that injects inert gas (e.g., Ar) bubbles into the molten Al, which then rises through the melt. The dissolved H_2_ in the melt diffuses in to the bubbles rising to the top of the melt [9]. Çolak et al. [33] compared the effects of fluxing and degassing on a secondary alloy Al-4Cu. The researcher used a reduced pressure test to evaluate the melt quality, i.e., by determining the difference between the densities of the samples solidified at normal atmospheres and samples solidified in a vacuum (80 mbar). After the density measurements, the density index (*DI*) can be calculated using Equation (1):

(1) $$DI=\frac{\rho_{1}-\rho_{2}}{\rho_{1}}\times 100$$

**Figure 2:**
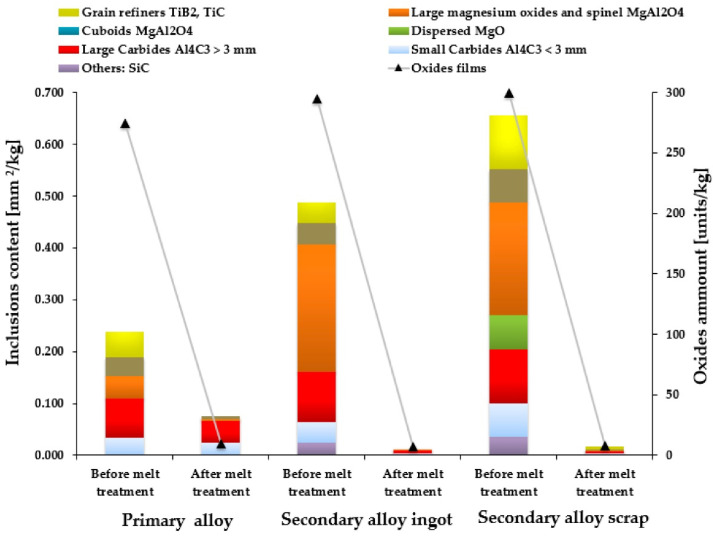
Quantification of the microinclusion and oxide films present in the alloys before and after the melt treatment [35].

$\rho_{1}$ is the density of a sample cast at 1 atm and $\rho_{2}$ is the density of a sample cast at the reduced pressure. The lower the *DI*, the less hydrogen is absorbed in the melt and the higher the molten quality. Normally, the index must be lower than 3% to proceed to the casting process [34]. Other parameters can be determined by observing the cross-section of these samples, including the bifilm index and the average pore area. The researchers observed that, with adequate preparation and optimal conditions (instruments’ preheating temperature, gas flow rate, and others), argon degassing obtains a higher melt quality and lower defects without the need of flux [33]. Bakedano et al. [35] were able to enhance the quality of two AlSi10MnMg(Fe) secondary alloys (one obtained from secondary ingots and the other from new scraps). The researchers obtained DI values such as the primary alloy with an adequate melt treatment, which consisted of deoxidation, degassing, and skimming by using the flux Elimoxal KF20 and a rotor impeller with Ar. With these treatments, it was also possible to reduce both macro and microinclusion. In the latter case, the inclusion content achieved after treatment was inferior to that of the primary alloy that was also treated, as shown in Figure 2. Considering this, Bakedano et al. concluded that it is feasible to produce structural components entirely from a secondary aluminum alloy. Thus, the performance of mechanical and microstructural analysis can add value to the study of these authors.

## 4. Aluminum Downcycling

Downcycling occurs when waste materials are processed, and their quality and characteristic are reduced and changed compared to the original [36]. The aluminum recycling system is a primary example of this phenomenon. With the mixing of different alloys and contamination with impurities, the SAA does not meet the strict chemical and mechanical property specification from specific applications, such as load-carrying automotive parts in the chassis or body [37]. The downcycling prevents closed-loop recycling, and the casting alloys end up being the last sink for the downcycled Al since they have a higher tolerance for impurities and require a more extensive alloying content, mainly silicon [20,38,39,40]. In addition, a bottleneck effect is created since certain sink alloys, such as AlSi9Cu3, can only be recycled into similar alloys due to their high alloying concentration [41]. Furthermore, there are three main categories of downcycling: functional, thermodynamic, and economic [36].

### 4.1. Functional Downcycling

**Figure 3:**
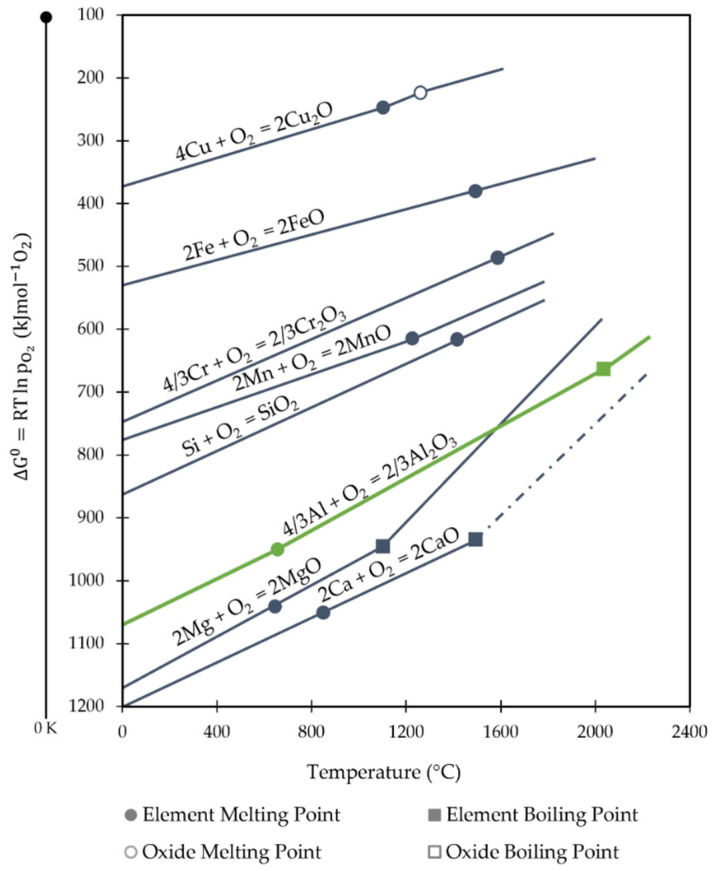
Ellingham diagram adapted from [45].

Functional downcycling, in which the material’s qualities are altered such that it can only be employed in less demanding applications, is the prominent downcycling phenomenon in Al recycling systems [36,42]. Al contaminated with different elements (e.g., copper and zinc) or nonmetallic inclusions is the primary cause of these downcycling rates, whereby the iron is the most hazardous contaminant [20]. Due to the high oxidation potential of Al, it is challenging to remove alloying elements with a simple oxidation method [36,43]. According to the Ellingham diagram, represented in Figure 3, only Ca and Mg may be removed since the other elements have higher free energy than aluminum and cannot be reduced into slag under the partial pressure of oxygen. A thermodynamic barrier to the removal of most elements is relatively high in the case of aluminum. Moreover, due to aluminum’s relatively low melting point and strong oxygen affinity, the degree to which other process parameters, such as temperature and flux composition, may be altered during remelting is considerably lower than it is for iron and copper production, further limiting additional removal processes [40,44].

#### Fe Contamination

In the Al-Si-Fe system, commonly used in Al casting, θ-AlFe is solidified first as the temperature decreases, formed as Al_3_Fe or Al_13_Fe_4_. This phase can dissolve up to 6% of Si, which in this case is referred to θ-AlFe(Si). Further solidification with a solute distribution leads to the formation of different ternary phases depending on the chemical composition of the alloys. These phases can be α-AlFeSi, γ-AlFeSi, or τ_2_-AFeSi. The α-AlFeSi forms when the Fe + Si content in the alloys is lower than 30 wt.%. Since this concentration does not exceed further, α is the ternary phase usually present in this case. This phase tends to form around the needles of θ-AlFe with a somewhat irregular shape. Moreover, if the Si/Fe ratio is higher after the solidification of the α-AlFeSi, the formation of another phase of β-AlFeSi occurs in high quantities. In equilibrium, the θ-AlFe, α-AlFeSi, and Si phases also transform into β-AlFeSi. The range of Si and Fe concentrations for the phases θ-AlFe(Si), α-, and β-AlFeSi is described in Table 1 [46].

The mechanical properties and microstructure of the Al alloys are affected by these phases in a number of ways. These intermetallic phases, which are very fragile and hard, decrease the SAA’s ductility while also increasing its hardness. The platelet or needle shapes of the β phases increase the stress concentration and the alloys’ general brittleness. These needles can increase porosity by preventing the feeding of liquid metal between them [47].

Studying additional systems, such as Al-Si-Mn(Fe) and Al-Si-Mg(Fe), is important since Al casting alloys typically contain other alloying elements.

The additions of Mn are commonly made to change the β phase to α by altering the (Fe, Mn)/Si ratio. Since Fe and Mn have similar atomic sizes, they behave similarly [48]. Ji et al. [49] reported that the Mn/Fe ratio must be lower than 0.5 to surpass the formation of the β phase in die casting. Moreover, the Fe-rich phase morphology changes from the needle-like morphology of β to the Chinese-script or polyhedral morphology. Some researchers claim that Mn additions improve the mechanical properties of the Fe-bearing alloys, but others consider them to be contaminations originating from the recycling process [50]. Hwang et al. [51] concluded that with 0.65 wt.% Mn addition to AlSi7Cu3.5Fe0.5, both ultimate tensile strength (UTS) and elongation increased, while yield strength (YS) did not change considerably. However, the UTS increased only with the additional application of heat treatment T6, while the elongation was maximum of 0.45%, which is still relatively low. The Mn additions also caused an increase in the volume of these sludge particles from 1.8% to 2.9%.

In the Al-Si-Fe-Mg system, for example, the AlSi7Mg0.3(Fe) alloy, in relation to the Fe-rich intermetallic the π-Al_8_FeMg_3_Si_6_ phases forms with a Chinese-script morphology. If the Fe content is about 0.1 wt.%, the π phase makes almost all the intermetallic compound content. With increasing Fe, the formation of the β phase is promoted, and the mechanical properties decrease. With 0.3 wt.% Fe, the π phase starts to form again on the surface of β phases with a Chinese script-like morphology. This layered structure of the π phase wrapped around lamellar β phases enhances the adhesive strength between the β and the matrix. The different phases have different effects on the crack propagation path. The π phase tends to show micro-cracks perpendicular to the tensile stress direction. In alloys with 0.2 wt.% Fe, the crack path tends to propagate at the interface between the β phase and the matrix. Moreover, cracks do not occur at this interface with the layered structure in the alloy with 0.3 wt.% Fe. Instead, the microcracks initiate at the π-phase interface and propagate through both phases. Thus, by promoting the formation of minor dimples during tensile stress, the UTS, YS, and elongation of alloys were enhanced (167 MPa, 75 MPa, and 6.0%, respectively) when compared with the alloy with 0.2 wt.% Fe (151 MPa, 69 MPa, and 5.4%, respectively). However, these values were lower than the alloy with only 1 wt.% Fe (186 MPa, 80 MPa, and 7.7%, respectively) [52].

### 4.2. Thermodynamic Downcycling

Thermodynamic downcycling occurs when an increase in thermodynamic effort is required to reprocess the material [36]. However, the primary processing techniques used to remove contaminants in Al, such as chlorination, electrolysis, and sedimentation, need significant energy consumption [53]. Therefore, Dhinakar et al. [54] explored the use of the sedimentation treatment to remove Fe-rich phases in the AlSi7Mg0.3(Fe) alloy with about 1 wt.% Fe. First, 1 wt% Mn and 1 wt% Cr were added to this alloy to enhance precipitation and were held at 620 °C for one hour. During this, the intermetallic phases precipitated and settled. Finally, the top layer of the molten aluminum was then poured out. With this technique, the researchers achieved a 77% yield rate of purified aluminum, but still had about 0.22 wt.% of Fe in the treated alloy. Therefore, even if some Fe is removed by sedimentation, it requires a significant amount of energy to hold the melt for one hour while creating a residue—the bottom sediments—that has a minimal chance of being recycled.

Another example of thermodynamic downcycling in the Al recycling system is the necessity to dilute the SAA with primary alloys to minimize the alloying concentration. Nevertheless, this diluting approach offers a thermodynamic degradation due to the energy-intensive nature of Al primary production [36,53]. The dilution procedure is mainly carried out to produce wrought alloys from mixed scrap, which necessitate a significant dilution to achieve the low alloying requirements [39].

### 4.3. Economic Downcycling

In the current recycling system, the downgrading of Al is economically attractive since costly separation is not required, and any expensive primary material for dilution is not added to SAA casting. Nevertheless, this depends on the strong demand for secondary castings, particularly in the automotive sector, where the amount of casting alloy required exceeds the amount of scrap produced. Therefore, the long-term viability of this arrangement may not be feasible. However, with the increased quality bottleneck of alloys used in the automobile industry and the introduction of electric vehicles, a scrap surplus (SS) may be achieved [21,53].

Cars are primarily associated with a quality bottleneck. Casting alloys, which are frequently SAAs and have a high percentage of other elements, are mixed with wrought alloys, which have minimal alloying content in the Al scraps from automobile shredders. The alloys created with these scraps can only be used as SAAs for casting since different types of alloys were mixed from shredding. Due to the SAA castings in the automotive sector already being primary recipients of recycled aluminum from all sectors, it will imply a discrepancy between secondary casting needs and the amount of scrap available, causing a SS [53].

According to van den Eynde et al. [39] and their material flow analysis model, the SS will begin to emerge over the next few years and will increase to 5.4 million tons in 2030 and 8.7 million tons in 2040. Additionally, Løvik et al. [20] anticipate that other nations such as China and Japan will initially absorb the regional SS of the USA and Europe. In the long term, these four regions will all exceed scraps by 2050. Although it can postpone the SS, the rising population and automobile ownership will result in a more significant SS. However, prolonging the product’s lifespan can reduce long-term excesses and slightly postpone the SS [53]. The demand for conventional casting alloy applications, such as engine blocks, is anticipated to decline due to the shift to electric vehicles, with the endorsement by the European Union and other nations to end the sale of fossil-fueled vehicles as soon as 2030—thus further aggravating the SS [20,38,55]. The inability to recycle some scraps will cause an increase in the production of primary aluminum, which has a highly negative impact on the economy and the environment, or can mean that up to 3.6 million tons of scrap will become “dead metal” in 2040 if no new recycling routes are created [38,39,53].

However, even though the downgrading of Al is still economically appealing, economic downcycling is created because the value of the material or the Al product is reduced and is expected to generate unrecyclable scrap [36]. This factor is critical in the automobile industry since casting production relies on cost reduction, yet high-quality casting is required to keep overall competitiveness [6].

### 4.4. Prospects for Al Downcycling

**Figure 4:**
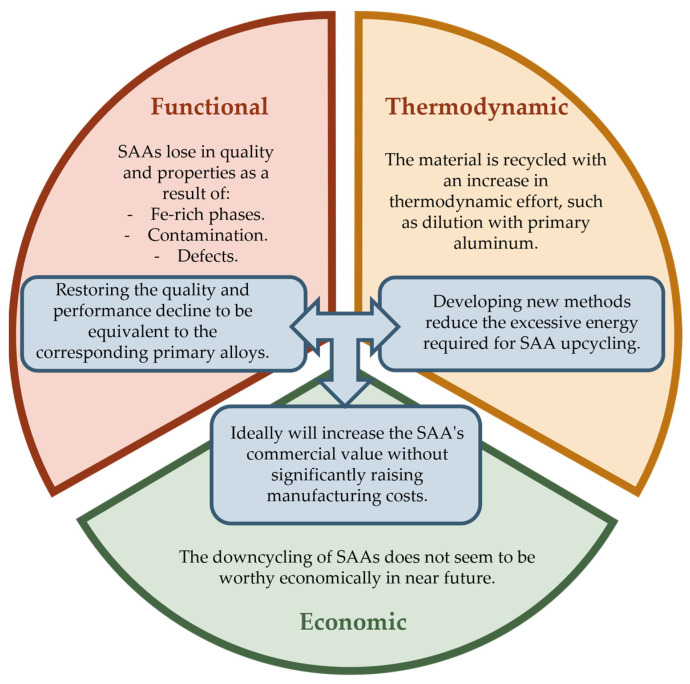
The prospective of the Al downcycling approach.

In this section, the three main aspects of Al downcycling are discussed: a reduction in properties mainly due to Fe-rich phases, an increase in energy consumption, and subsequently a reduction in the commercial value of SAAs. It was also recognized that recycling processes will continue to be economically advantageous in the foreseeable future. The authors present a scheme as illustrated in Figure 4, showing the main problems and possible solutions to achieve upcycling of SAAs. The general applicability of these alloys may change as a result of the ongoing production of SAAs for casting applications and the transition of combustive automotive industry to electric cars may alter the overall demand for these kinds of alloys and result in an excessive amount of scrap material that is unsuitable for use. To preserve the automobile industry’s competitive economic edge, it is crucial to develop techniques of reversing the functional downcycling by achieving alloys with similar qualities and properties as primaries ones, without significantly increasing the thermodynamical efforts. This will lead to an overall increase in the SAA values and a reduction in the economic downcycling.

## 5. The Microstructure and Mechanical Performance of SAAs

Gravity casting, HPDC, and other casting processes use a number of SAAs with different chemical compositions, some of which are presented in Table 2. Particularly in the automotive sectors, SAAs are employed to produce engine blocks and cylinder heads made of AlSi9Cu3(Fe) alloys, while wheels and bellhousing are made of AlSi7Mg0.3 alloys.

**Figure 5:**
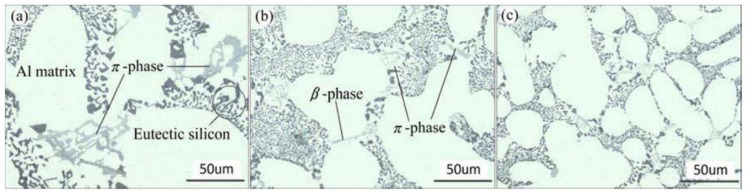
AlSi7Mg0.3 with 0.15 wt.% Fe sand casting at different cooling rate: (**a**) 0.19 °C/s; (**b**) 0.65 °C/s; and (**c**) 6.25 °C/s. Reproduced from [57] with permission from Elsevier, 2022.

The casting process and the chemical composition have a significant effect on the microstructure and on the mechanical performance of the alloys. In the gravity sand casting process, the AlSi7Mg0.3 alloy is normally used. The typical microstructure of this alloy is mainly composed of α-Al dendrites with the eutectic Si constituent distributed in the interdendritic spacing in the Al matrix. As seen in Figure 5, the eutectic Si particles present a fibrous-like shape caused by the modification procedure carried out during casting by adding Sr. Since the AlSi7Mg0.3 alloy contains Mg, the Fe-rich phases are mainly β and π. Chen et al. [57] studied the effect of the cooling rate on the microstructure of AlSi7Mg0.3 with 0.15 wt.% Fe produced by gravity sand casting. The microstructure corresponding to cooling rates of 0.19, 0.65, and 6.25 °C/s is presented in Figure 5. It was observed that with an increase in the cooling rate, the secondary dendrite arm spacing (SDAS) significantly decreased from almost 68 µm to 20 µm. Higher cooling rates led to bigger undercoolings which promoted the nucleation of α-Al and a refinement of this phase and Si particles. Regarding the Fe intermetallic compounds, it was observed that these phases form between the eutectic Si and the α-Al dendrites. Figure 5a shows that the morphology of the π-Al8FeMg3Si6 phase typically appears in a Chinese-script morphology, whereas Figure 5b illustrates the needle-shaped β-Al5FeSi phase. Moreover, the increasing cooling rate also refined the intermetallic phases, from almost 28 to 18 µm.

**Figure 6:**
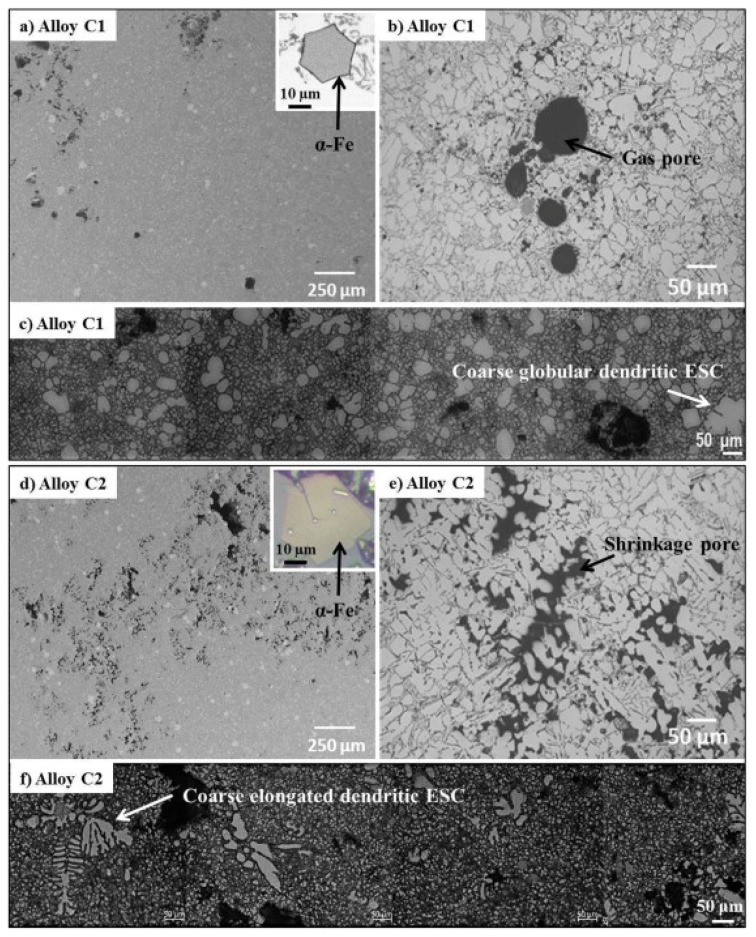
Commercial SAA AlSi9Cu3(Fe) microstructure. (**a**,**b**) C1 with 0.9 wt.% Fe and (**d**–**f**) C2 with 4.2 wt.% Fe. Reprinted with permission from [58]. 2022, Elsevier.

The microstructure of a commercial AlSi9Cu3 type with two distinct Fe concentrations, 0.9 and 4.2 wt% (referred to as C1 and C2, respectively), was examined by Reyes et al. [58]. The α-Al phase structure is similar between the two alloys (Figure 6) and consists of fine dendritic grains due to high cooling rates. However, the main differences are the morphology of the external solidified crystal (ESC) dendrites (globular and elongated), the population of the α-Fe-rich phase attributed to the Fe%, and the gas porosities and shrinkage porosities. These α-grains have a coarse spherical dendritic structure in low Fe contents (Figure 6c), whereas they tend to elongate when the Fe content rises (Figure 6f). In addition, the often-occurring Fe-rich phase in the HPDC cast AlSi9Cu3 alloy is a hexagonal-shaped α phase. The Fe-rich alloy contains an α phase with more irregular forms and tiny spherical particles, such as non-metallic inclusions, embedded therein. These inclusions may facilitate fragile fractures through the particle. The existence of a thin needle-shaped β phase, whose development is aided by an excess of Fe, was also detected in the sample after it had been etched, according to the research. Additionally, by hindering interdendritic feeding, the growth of Fe-rich phases in the C2 alloy enhanced the existence of shrinkage porosity.

**Figure 7:**
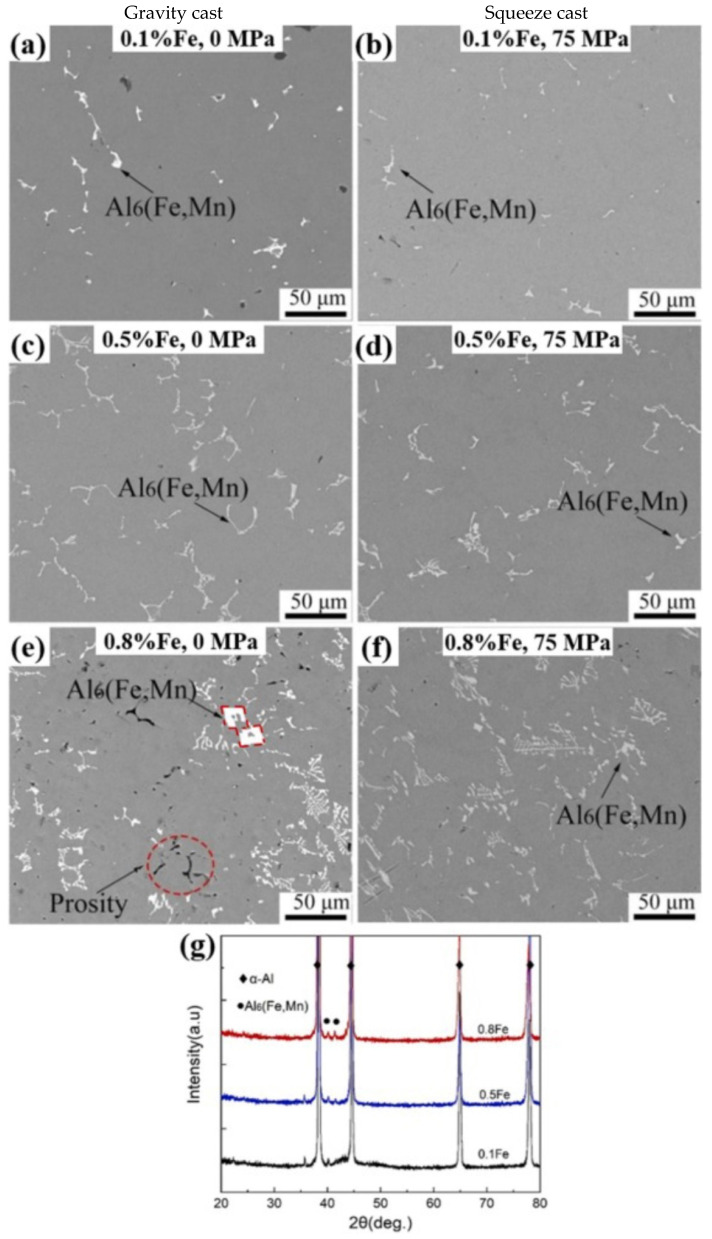
BSE-SEM images of the gravity (0 MPa) and squeeze-cast (75 MPa) AlMg3Mn1(Fe) alloy with different Fe contents—(**a**,**b**) 0.1%; (**c**,**d**) 0.5%; and (**e**,**f**) 0.8% Fe. (**g**) XRD spectrum of the gravity-casted alloy. Reproduced from [59] with permission from Elsevier, 2022.

Zhao et al. investigated the effect of Fe content on the evolution of intermetallic phases in the gravity- and squeeze-cast AlMg3Mn1(Fe) alloys. Al_6_(FeMn) is the Fe-rich phase that is observed in this alloy since Si is not present. Figure 7 illustrates how the Chinese-script morphology becomes more evident with the increase in Fe. Thus, other morphologies, including rhombus (which resembles a hollow rectangular prism) and plate-like morphologies, tend to be reduced as a result of various solidification stages. An applied pressure of about 75 MPa was responsible for enhancing the Mg solubility in α-Al and any Mg was not identified in the Fe-rich phases. This was confirmed by XRD analysis as shown in Figure 7g, revealing the presence of Al_6_(FeMn). Moreover, the change in mechanical properties from 0.1 to 0.8 wt% of Fe was significant. Along with an increase in Fe, UTS, YS, and hardness rates typically rise as well. The Brinell hardness increased from 64 to 77 HB, UTS rose from 244 to 289 MPa, and YS increased by roughly 20% from 122 to 146 MPa. However, the elongation values, which decreased from 34% to 12%, show the biggest change of nearly 65%. The decrease in ductility can be associated with both the increase in hard brittle intermetallic phases and also with the increase in porosity and pore diameters, which both function as initiation sites for microcracks [59].

The mechanical properties of the two most popular SSAs for gravity casting and HPDC, AlSi7Mg0.3 and AlSi9Cu3(Fe), in their as-cast states are shown in Table 3, respectively. As a general point of view, tensile properties can be deteriorated when the Fe content rises over the limit set by the NP EN 1706 standard. As seen in Table 3, some authors revealed that the cast with a Fe% less than 0.15, the limit defined by the standard, behaves in the scope of standard; however, following an increase of 0.05% in Fe above that limit, the properties decreased, though they did not considerably deteriorate following a further increase in Fe. This table also presents the data of a similar alloy produced by low-pressure die casting (LPDC), which had significantly better UTS, YS, and elongation values than the standard and the alloys produced by gravity casting. This improvement can be attributed to the melt quality and highly efficient feeding capabilities of this method.

Regarding the mechanical properties of AlSi9Cu3(Fe), i.e., another SAA mostly used in HPDC, it can be seen that almost all properties are in the scope of the standard, and that the elongation is expected to be very small. However, the properties are influenced by the processing conditions, mainly pressure.

## 6. Valorization of SAAs

As a result of the cascade recycling system of the Al alloys and the anticipated increase in scrap excess, it is necessary to explore and develop methods to increase the value of SAAs by limiting the impacts of Fe-rich phases. Some strategies for achieving these goals include creating new alloy systems, applying different melt treatments such as ultrasonic waves, and trying to control the morphology and size of the phases through heat treatments.

### 6.1. New Aluminum Alloy Systems

Considering that the chemical composition significantly impacts the microstructure and mechanical properties of Al alloys, developing new systems for Al alloys may be one method to increase the use and value of SAA. The principle consists of modifying the type or morphology (or both) of Fe-rich phases by adding other elements, customarily called “neutralizing elements”. Numerous element additions have been studied recently and were established as a helpful strategy to prevent various adverse effects of Fe-rich phases [20,53,65]. Table 4 presents an overview of the most recent works which aim to modify Fe phases through chemical additions. The most studied addition was Cr, leading to an increase in Fe-rich phase fraction and the transformation of the β and π phases into smaller α phases. This transformation occurs because Fe, Mn, and Cr can substitute one another in the body-centered cubic crystal structure of the α phase, thus altering the (Fe, Mn, Cr)/Si ratio, commonly represented as Al_15_(Fe, Mn, Cr)_3_Si_2_. The high specific gravity of the particles causes them to settle to the bottom of the melt on holding furnaces and are referred to as sludge. The tendency of these phases to form can be predicted with simple calculations of the *sludge factor* (*SF*) which is calculated by Equation (2) in the case of the Al-Si-Cu alloys:

(2) SF=wt.%Fe+2∗wt.%Mn+3∗wt.%Cr

In the foundry, the SF value is typically not higher than 1.7. However, this law is arbitrary and vague [50,66].

Several studies evaluated the effect that increasing the Cr content has on the alloys. With additions ranging between 0.5 and 1.5% of Cr, Bolibruchová et al. [67] continued to detect very lengthy and thick β phases. In this manner, the additions did not improve the elongation despite achieving the maximum UTS values for 1 wt% Cr. Thus, Cr can be defined as impurities alongside Fe and Mn. The Co and Mo additions were two of the most promising additions since they could avoid the formation of the needle-like β phase. However, other additions, such as Ni and Li, have little impact on these intermetallic phases. More recently, several researchers have studied the effect of microalloying rare earth elements (REEs) on Al alloys with promising results, which will be discussed further.

As a conclusion, when analyzing most of these investigations, the relationships between the microstructural and Fe-rich phase morphology modifications with improvements in mechanical characteristics, particularly elongation at break, are critical knowledge gaps. Furthermore, additional investigations are needed to establish chemical modifications as a viable industrial route for the valorization of SAAs.

**Table 4:** An overview of elemental additions to SAAs in recent investigations processed by casting.

Element	Alloy	Fe (wt.%)	Mn/Fe	Observations	Reference
Be	AlSi7Mg0.3	Up to 0.7	~0.02	A small addition (500 ppm) of Be provoked the transformation of π into the β phase. Even though Be could avoid the effects of Fe phases on Al alloys, the researchers did not promote the use of these additions due to the toxic nature of Be.	[68,69]
Co	AlSi7Mg0.3	Up to 1.0	-	It was shown that Co/Fe ratios between 1 and 2 are ideal for intermetallic compounds with a Chinese-script shape.	[70]
Cr	AlSi3Mg0.6	0.1	~1.0	The addition of Cr and Mn provoked the intermetallic phase shape to modify the needle-like morphology of the β and π phase to a more rounded α phase.	[71]
	AlSi7Mg0.3	1.7	0.3	High Cr additions caused the β phase to become very long and thick, without significantly improving the mechanical properties of the alloy.	[72]
	AlSi9Cu3	0.8	0.6	Cr addition up to 0.12 wt.% alongside a high cooling rate from HPDC caused the formation of α-Al_x_(Fe,Mn,Cr)_y_Si_z_. The Fe-rich intermetallic compounds’ volume fraction and size increased as the Cr content increased.	[73]
	AlSi9Cu3	4.0	~0.1	A greater quantity of cubic α phase made up of several small particles results with the addition of Cr. Cr concentration gradients occurred in the cubic α phase with the Al_13_Cr_4_Si_4_ phase at the center of the particles.	[74]
	AlSi20	5.0	-	High quantities of Cr additions (5%) achieved hardness above 200 HVN. This is due to Al_3_FeSi_2_ transforming to a more complex dendritic-shaped σ-CrFe compound.	[75]
Li	AlSi7Mg0.3	0.1	0.5	Li additions improved the hardness of the alloy due to the precipitation of AlLiSi phases in the Al matrix (HV increased between 10 and 25 depending on the cooling rate). However, other phases, such as β, occurred as the base alloy.	[76]
Mo	AlSi6Cu3	0.3 and 0.7	-	In the alloys with low Fe content, it was possible to avoid β-phase precipitation with only 0.24% Mo addition. Moreover, in the high-Fe alloy, adding 0.41% was needed suppress the β-phase formation.	[77]
	AlSi6Cu3	0.7	0.4	Due to the lower solubility of Mo in Al, it was easier to form Al-Mo intermetallic than when adding Mn. Thus, Mn and Mo additions combined could achieve a better modification of the β phase.	[77]
Ni	AlSi7Mg0.3	1.3	0.07	Ni additions did not modify the β-phase morphology, without having any significant effect on tensile properties.	[78]
	AlSi6Cu4	0.8	0.7	Ni addition thickened not only the needed-like β phase but also the α phase.	[67]
Ti	AlSi20	5.0	-	With the addition of Ti, the significant Fe phase was the Al_3_FeSi_2_ with a plaque morphology. Additions of around 1 wt.% Ti formed Ti_5_Si_3_ during the acicular phase. While additions above 5 wt.% caused the segregation of eutectic Si around this binary intermetallic, the hardness increased from 106 to 144 HV.	[75]
Zn	AlSi9Cu3Mg0.3	0.2	-	The Zn addition with the Sr eutectic modification helped to modify the morphology of the Fe-rich phases. The particle size was also reduced by almost half. With 0.58% Zn additions, thin and long β phases were still detected. The tensile properties of UTS and elongation were improved, but not YS.	[79]

#### Rare Earth Element Microalloying

REE additions do not exceed 1% in weight to avoid the formation of excessive Al-REE phases, which tend to be detrimental to the mechanical properties of the alloys. Thus, usually, it is referred to as REE microalloying. In addition, for example, La, Ce, and Er were proven to refine the α-Al grains, modify the morphology of the eutectic Si, and improve fluidity. With this alteration, the mechanical properties, in general, were enhanced [80,81,82].

The most studied REE addition in aluminum alloys is La. For example, Liu et al. [83] studied various additions of La to the AlSi7Cu3Mg0.3Mn0.3-Fe0.4 casting alloy. The researcher reported that combining La microalloying with conventional refinement treatment with Al-Ti-B master alloy boosted the refinement. The lowest secondary dendrite arm spacing (SDAS) of roughly 12 µm was achieved using Al-Ti-B and La addition. The low solubility of La in α-Al allows the La to be concentrated at the interface of α-Al/liquid during solidification, thus causing constitutional supercooling and limiting grain growth. Despite this, the UTS was similar in all conditions. With values close to 260 MPa, the elongation was greatly improved. Al-Ti-B + 0.1La additions increased the elongation values by around 70% from 2.5% of the base alloy. These additions also caused the modification of sheet-like eutectic Si to fibrous and the transformation of β-Fe to α-Fe, thus improving the alloy’s ductility even though one of the primary causes of fracture was still the presence of Fe-rich phases. Furthermore, Li et al. [84] significantly enhanced the mechanical properties of a hypereutectic Al-Si casting alloy with 0.8 wt% Fe by adding 0.5 wt% La and 3.6 wt% Mg. The needle-like β phase changed into a script-like π phase by these chemical modifications. During the solution heat treatment, the latter phase was broken down into small and granular-like particles. This heat treatment significantly improved the mechanical properties, with the UTS rising from 140 to 290 MPa with modification, then to 406 MPa with T6, and elongation rising from 1.27% to 2.75% before reaching the maximum of 4.28 with T6.

REE microalloying has also been proven to reduce casting defects, such as hot tearing. This discontinuity is one of the most significant problems in aluminum foundry industries when a crack is visible on the casting surface at the end of solidification. These defects tend to originate at temperatures of the mushy zone of the alloys and when the feeding is insufficient, leading to strains in the remaining liquid formation and causing cracks [85]. Tao et al. [86], with additions of 0.15 wt.% Y, enhanced the hot tearing resistance of the AlCu4.4Mg1.5Zr0.15 alloy. These additions reduce the solidification temperature range and create minor shrinkage strains, thus reducing the probability of hot tearing.

### 6.2. Ultrasonic Melt Treatment

Ultrasonic melt treatment (UST) has been thoroughly proven to enhance the properties and quality of Al castings. The UST can refine α-Al grains, change the eutectic phases, and degas the melt by causing cavitation and acoustic streaming. Sound waves (frequency ~20 kHz) are spread throughout the melt by acoustic streaming. Metals can be mixed and stirred using this mechanism rather than mechanical stirring. On the other hand, cavitation corresponds to several stages of tiny bubbles through the melt, such as formation, growth, pulsation, and collapse [87].

Grilo et al. [88] evaluated the effect of the UST at different melt temperatures. The temperature range studied varied from 640 to 700 °C, and an ultrasonic transducer with 1.6 W/cm^2^ and 20.1 ± 0.25 kHz frequency was employed in the AlSi7Mg alloy. Lower treatment temperatures (about 640 °C) promoted the formation of rounder grains, often known as rosette-like morphology, and lower porosity levels. The grain size decreased by nearly 66% at this temperature, reaching its smallest value of 399 ± 84 µm. Despite this, intermetallic phases, including Mg_2_Si, β, and π, were found in the microstructure without appreciable morphological modifications.

**Figure 8:**
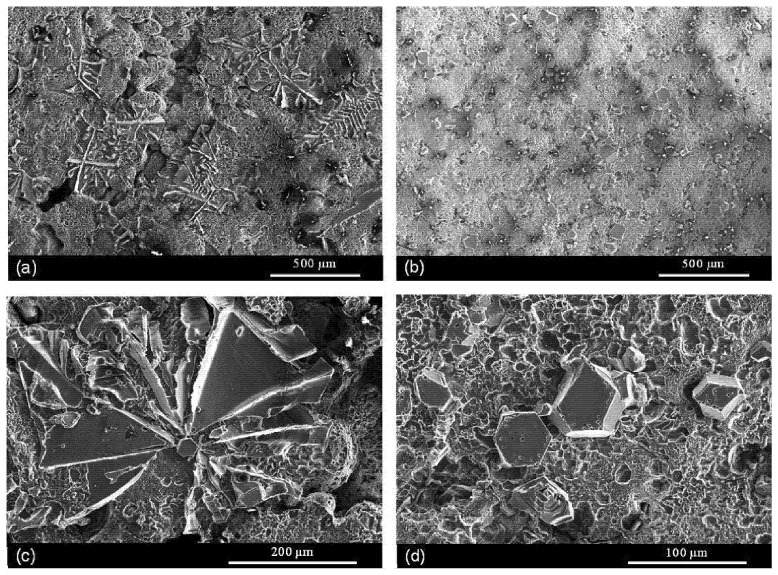
Deep-etched microstructure of the α-Al_15_(Fe, Mn)_3_Si_2_ intermetallic formed: (**a**,**c**) without UST and (**b**,**d**) with ultrasonication. Reproduced from [89] with permission from Elsevier, 2022.

Kotadia et al. [89], in a similar study but with a specific focus on the AlSi2Mg2Fe1.2Mn0.5 alloy, observed a transformation of the coarse Chinese-script α-Al_15_(Fe,Mn)_3_Si_2_ (Figure 8a) to refined polygonal particles (Figure 8b) when a UST was applied. The researchers suggested that the UST can promote the α phase nucleation and the solute homogenization at the solid–liquid interfaces. This homogenization impends the radial branching from the central polygonal particle, i.e., the formation of a Chinese-script morphology.

Another study [90] defined two critical temperature ranges for UST in the formation and growth of intermetallic phases on the AlSi12Cu3Mg0.35Zn2Ni0.3 (Fe1.3, Mn0.4, Cr0.15) alloy. The application of UST before the precipitation of Fe-rich phases (750 °C–850 °C) enhances the nucleation of these particles and reduces their size. However, the morphology is not modified and appears to be branched. On the other hand, if the UST is applied during precipitation temperatures ranging from 600 °C to 750 °C, the size is diminished, but the particles can also be broken into smaller polyhedral shapes. Due to the relatively low UST time, around 4 min, these smaller particles tend to remain agglomerated.

### 6.3. Heat Treatment

Heat treatments are frequently used in various industries to improve the mechanical properties of Al castings. Although T4 (solution treatment and natural aging) and T7 (solution treatment and artificially over-aging) are employed, T6 (solution treatment and artificially aging) is the heat treatment that is most frequently used [91]. Eva et al. [92] evaluated the effects of solution treatment conditions on the secondary AlSi9Cu3 alloy with about 0.8 wt.% Fe. After being sand cast, the alloys underwent T4 heat treatment at various solution treatment temperatures (505 °C, 515 °C, and 525 °C) and times (2, 4, 8, 16, and 32 h). The study then assessed how the Fe-rich phases evolved morphologically. The β phase tended to evolve to a very thin needle shape, becoming hard to detect. The α-Al_15_(Fe, Mn)_3_Si_2_ lost its Chinese-script morphology with treatments at 505 °C by fragmenting the different branches. Rising the temperature to 515 °C allowed some of these phases to dissolve. With a solution treatment at 515 °C for 4 h, the Fe-rich phases fraction decreased by about 67% from the 4.8% fraction of the non-heat-treated alloy. Increasing the holding time did not significantly change the fraction of these phases. Thus, the solution treatment temperature has a more significant effect on the Fe-rich phases than the holding time.

### 6.4. Achivements and Future Challenges

**Figure 9:**
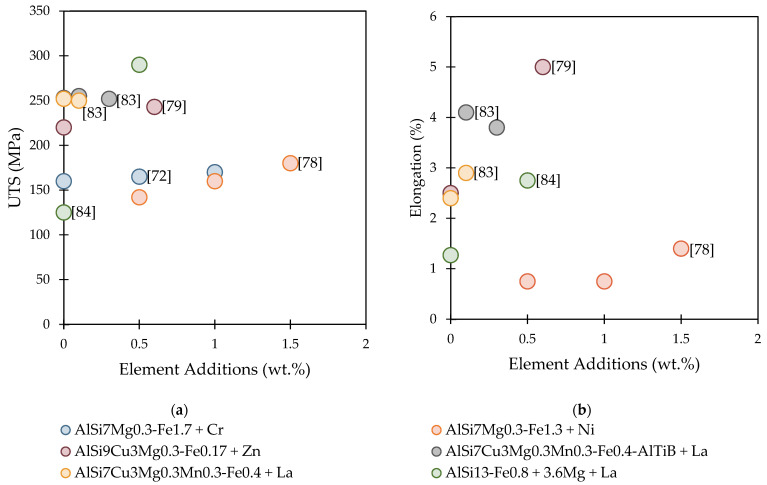
A summary of the improvements in mechanical properties obtained from the researches presented in Table 4: (**a**) UTS and (**b**) elongation.

This section reviewed a number of research studies that sought to alter the morphology of the Fe-rich phases. The changed alloys were only mechanically characterized in five of these investigations. A summary of the UTS and elongation values obtained from different authors is shown in Figure 9. Comparing the UTS values showed that the increase in element additions did not significantly improve in most studies, except in the case of the combined addition of 3.6 wt.% Mg and 0.5 wt.% La. On the other hand, the elongation values were efficiently increased in various other studies, especially when REEs were used.

Furthermore, the studies in the application of UST did not present a mechanical characterization, and thus it may be interesting to evaluate the effect of morphology on the mechanical properties of the alloys when using UST.

Therefore, microalloying with rare earth is a promising procedure to revert the functional downcycling of SAAs. However, some challenges must be tackled, such as determining the optimal concentration to enhance the mechanical properties and understanding how the elements act as neutralizers in the Fe-rich phases. Moreover, it is of relevance to observe the results of combining the different methods and to discuss which one alone can improve different aspects of the overall alloy qualities.

## 7. Conclusions

The expected higher demand for Al in the market in the near future alongside the need to move towards a more circular economy and lowering the dependence on primary Al is needed to prompt the production of SAAs. However, the current Al recycling approaches suffer from different types of downcycling that prevent the wider use of SAAs. The loss of quality and properties due to Fe contamination is the main factor of functional downcycling. It has been extensively proven that hard and fragile Fe-rich phases lower the ductility of the Al alloys and the applicability in structural applications.

The Al recycling system is comprised of many steps that may cause changes in the quality of the alloys. The scrap is first sorted and compacted to facilitate handling. Oxides, Fe, and other metals can contaminate Al if the sorting is conducted incorrectly. Then, the remelting of Al scraps is proceeded in reverberatory or tilt rotary furnaces. However, the two primary factors that might adversely impact the chemical composition, microstructure, and mechanical properties of the alloys are the number of recycling cycles and the percentage of returnable materials. Afterwards, the liquid SAAs are refined by degassing and fluxing processes to reduce the amounts of impurities that can cause defects and porosities in final casts.

Al downcycling is still economically favorable due to the reduction in costs in raw materials and energy. However, function and thermodynamic downcycling approaches are the two main reasons for a reduction in the value of SAAs. The primary factor contributing to this loss is the reduction in mechanical properties brought about by an increase in defects and Fe-rich phases. Moreover, the considerable energy required to fully recover the SAAs into primary alloys is attributed to the thermodynamical approach. To improve the competitive edge of the industries, it is therefore vital to find new methods such as upcycling approaches that require less energy consumption.

AlSi7Mg0.3 and AlSi9Cu3 are the two SAA alloys from Al-Si-(Fe) systems mostly used for gravity and high-pressure casting, respectively. The microstructures of these two alloys are similar and constituted by the dendrites of α-Al, given that the matrix and the eutectic constituent of Si particles solidified at the interdendritic spaces. The Fe-rich phases are presented mainly with needle-shape morphologies and as hexagonal shapes for HPDC parts. The mechanical properties of these alloys are relatively degraded in the presence of Fe. This reduction is highlighted in HPDC parts that can contain a Fe content up to 1.3 wt.%, causing a low elongation for almost 1%.

Various efforts have been made to develop technologies to add value to the SSAs such as elemental additions or ultrasonic melt treatments. The addition of Mn and Cr to the Al melt is the most studied upcycling approach. However, some authors consider these elements as impurities since they tend to enhance the Fe-rich phase formation, especially the sludge particles that tend to settle in the bottom of the melt. Other additions such as Ni and Li showed no improvement. The most promising additions examined in the literature were the rare earth elements in low quantities. Additions of La were not only proven to enhance the ductility of the Al alloys but also to reduce defects on the alloys, such as hot tearing. Apart from elemental additions, the ultrasonic melt treatment alongside degassing the melt has also been carried out to modify the Fe-rich phases. The effects of this treatment were enhanced by treating the melt at temperatures between 600 °C and 750 °C, through which a small polygonal α phase is achieved alongside a Chinese-script morphology. Another noteworthy result of these studies is the modification of the β phase to α or π. However, these studies lack a robust mechanical characterization of the alloys with a deep understanding of the relationship between the Fe-rich phases and the mechanical performance.

## Figures and Tables

**Table 1:** Intermetallic phase concentration range (wt%) adapted from [46].

	θ-AlFe(Si)		α-AlFeSi		β-AlFeSi	
	Si	Fe	Si	Fe	Si	Fe
Non-equilibrium (as-cast state)	1–6	35–42	6–13	31–35	13–16	25–29
Equilibrium (heat-treated at 600 °C)	1–5	36–41	6–9.5	32–36	14–16	27–28
Example formula	Al_3_Fe or Al_13_Fe_4_		Al_8_Fe_2_Si		Al_5_FeSi	

**Table 2:** Chemical composition (in wt.%) of commonly used SAAs [56].

Alloy	Al	Si	Mg	Cu	Fe	Mn	Cr	Ti	Zn	Others
AlSi7Mg0.3(Fe) 356.0	90–93	6.5–7.5	0.2–0.45	<0.25	<0.6	<0.35	-	<0.25	<0.35	<0.15
AlSi9Cu(Fe) A308	80–90	8–11	0.05–0.55	2–4	<1.3	<0.55	<0.15	<0.25	<1.2	Ni, Pb, Si, Sn
AlSi12 413.0	82–89	11–13	<0.1	<1	<2	<0.35	-	-	<0.5	<0.25
AlZn3Mg1(Fe) 705.0	92–95	<0.2	1.4–1.8	<0.2	<0.8	0.4–0.6	0.2–0.4	<0.25	2.7–3.3	<0.15

**Table 3:** The mechanical properties of primary and secondary casting alloys.

Alloy	Class	Process	Fe (wt.%)	UTS (MPa)	YS (MPa)	A (%)	Reference
AlSi7Mg0.3	NP EN 1706	Gravity	<0.15	min. 170	min. 90	min. 2.5	[60]
	Primary alloy	LPDC	~0.1	304 ± 8	229 ± 8	11 ± 3	[61]
		Gravity	0.09	186	80	8	[52]
	Secondary alloy	Gravity	0.2	151	69	5	
			0.33	167	75	6	
AlSi9Cu3(Fe)	NP EN 1706	HPDC	0.6–1.1	min. 240	min. 140	min. 1	[60]
	Secondary alloy	HPDC	0.8	323	252	3.8	[62]
			0.8	~200	152	1	[63]
			1.1	262 ± 3	158 ± 4	2 ± 0.1	[64]

## Data Availability

Not applicable.
